# Comparative outcomes of patients with esophageal cancer undergoing single-port and four-port thoracoscopic esophagectomy: a single-center experience

**DOI:** 10.1007/s13304-025-02244-8

**Published:** 2025-06-23

**Authors:** Xiaobo Chen, Yanan Bao, Yue Cui, Qinghua Zhou, Haoran Chi, Xinghe Tong, Xiaoxing Wang, Yunping Zhao

**Affiliations:** 1 Department of Lung Cancer Center, West China Hospital of Sichuan University, Chengdu, 610000 Sichuan China; 2 Department of Thoracic Surgery, The First Affiliated Hospital of Kunming Medical University, Kunming, 650000 Yunnan China

**Keywords:** Esophageal cancer, Single-port thoracoscopic esophagectomy, Four-port thoracoscopic esophagectomy, Outcome, Complications, Intractable pain

## Abstract

We aimed to compare the outcomes and postoperative complications of patients with esophageal cancer after single-port thoracoscopic esophagectomy (SPTE) and four-port thoracoscopic esophagectomy (FPTE). We retrospectively collected data from patients who underwent surgery at the First Affiliated Hospital of Kunming Medical University from February 2019 to November 2022. In total, 86 patients were included in the SPTE group and 114 patients were included in the FPTE group. Compared to patients undergoing FPTE, significantly shorter postoperative hospital stay (13.54 ± 3.88 vs. 19.08 ± 5.09 days), shorter chest drainage time (6.06 ± 2.16 vs. 7.45 ± 1.68 days), lower estimated intraoperative blood loss (226.05 ± 94.87 vs. 363.16 ± 78.92 mL), and longer overall length of surgery (346.34 ± 53.86 vs. 332.89 ± 28.86 min) and longer length of abdominal surgery (183.46 ± 33.33 vs. 166.05 ± 22.07 min) were observed among patients undergoing SPTE; however, no significant difference was observed regarding 3-month, 6-month, and 12-month overall survival, time to start oral feeding, and length of thoracic surgery. Furthermore, postoperative intractable pain lasting for 3 months (39.5% vs. 21.0%), pneumonia (34.2% vs. 11.6%), empyema (14.9% vs. 3.5%), and chylothorax (10.5% vs. 2.3%) were significantly more frequent in the FPTE group, whereas postoperative gastrointestinal symptoms (41.9% vs. 23.7%) were significantly more frequent in the SPTE group. There was no significant difference between the groups regarding postoperative arrhythmia, recurrent laryngeal nerve injury, *esophagotracheal* fistula, second surgery, and intractable pain lasting for 6 and 12 months. Compared to FPTE, SPTE may improve patients’ outcomes and prevent some of the major complications.

## Introduction

Worldwide, esophageal cancer is the seventh most common type of cancer and the sixth leading cause of mortality due to cancer [1]. Globally, over 600,000 people are diagnosed with esophageal cancer each year with a five-year survival rate of less than 20% [2]. In 2019, esophageal cancer led to more than 498,000 deaths and 11.7 million disability-adjusted life-years (DALYs) [3].

Even with the recent progress in the chemotherapy and immunotherapy of esophageal cancer, surgical intervention remains the primary treatment for esophageal cancer [4]. Despite recent advancements in surgical techniques and perioperative management, esophagectomy remains a highly specialized surgery with a high rate of complications, such as anastomotic leakage, pneumonia, atrial fibrillation, recurrent laryngeal nerve injury, and chylothorax [4]. Compared to minimally invasive esophagectomy, traditional open esophagectomy leads to significant trauma, intraoperative blood loss, and a high rate of perioperative complications [5]. Postoperative complications of surgical trauma are a significant clinical concern and greatly affect patients’ recovery [6]. For instance, a recent study reported that nearly 59% of patients undergoing esophagectomy experience at least one complication, with pneumonia and atrial dysrhythmia being the most common [7]. The concepts of precision surgery, minimally invasive surgery, and enhanced postoperative recovery have increasingly received attention in esophagectomy for esophageal cancer [8]. Minimally invasive techniques, especially single-port thoracoscopy have been reported to reduce the risk of postoperative complications and shorten hospitalization length [9]. A study with 75 patients undergoing uniprortal video-assisted thoracoscopic surgery (VATS) or open McKeown esophagectomy reported that re-operation for major complications was more frequent in the open group and chest drainage time and postoperative stay were shorter in the VATS group [10]. A multicenter study with 458 patients who underwent VATS lobectomy or anatomical segmentectomy reported that compared to multi-port approaches, single-incision thoracoscopic surgery leads to shorter chest tube drainage and postoperative hospitalization time, lower pain scores on the first and second postoperative days, longer operation time, and greater intraoperative blood loss but do not change morbidity, mortality, and pain score on the third postoperative days [11].

Currently, thoracoscopic resection of esophageal cancer is commonly performed using the"four-port technique", while reports on single-port thoracoscopic esophageal cancer resection are limited. In this study, we retrospectively analyzed the clinical data from patients who underwent McKeown single-port thoracoscopic esophagectomy (SPTE) and four-port thoracoscopic esophagectomy (FPTE) due to esophageal cancer at the First Affiliated Hospital of Kunming Medical University from February 2019 to November 2022. Particularly, we compared several outcome measures, such as overall survival and length of hospital stay, and several types of complications between patients with esophageal cancer undergoing SPTE and FPTE.

## Methods

### Patients

We retrospectively collected the clinical and pathological data of patients who underwent McKeown SPTE or FPTE at the First Affiliated Hospital of Kunming Medical University between February 2019 and November 2022. Patients with incomplete data or a history of previous thoracic surgery, gastrointestinal diseases, severe preoperative complications, metastasis, or concurrent organ resection were excluded from this study. All patients underwent multidisciplinary discussions before surgery. Tumor classification was conducted based on the 8 th edition TNM staging of the Union for International Cancer Control (UICC).

Two skilled surgeons proficient in open esophagectomy, and multi-port and single-port thoracoscopic esophageal cancer resection performed all surgeries. Additionally, the surgical procedures were supervised by a specialized professor with over 30 years of experience in esophageal surgery. This study was approved by the Ethics Committee of the First Affiliated Hospital of Kunming Medical University (Number: 20231214-56). Written informed consent for participation was waived by the Ethics committee of the First Affiliated Hospital of Kunming Medical University due to the retrospective nature of this study. We also adhered to the ethics standards defined by the revised Declaration of Helsinki.

### Preoperative evaluation and perioperative preparations

Complete gastroscopy and biopsy were conducted in the outpatient setting to confirm the diagnosis of esophageal cancer. Preoperative assessments were as follows: complete blood count, blood biochemical tests, tumor markers, pulmonary function tests, electrocardiography, echocardiography, enhanced chest and abdominal CT scan, upper gastrointestinal contrast studies, bone scan, and brain magnetic resonance imaging. Additional assessments, such as endoscopic ultrasound (EUS) and positron emission tomography (PET), were conducted if necessary. The preliminary clinical staging was determined as “locally advanced, non-metastatic esophageal adenocarcinoma and esophageal squamous cell carcinoma (stage T1b– T4, N1–N3, M0)”, needing resection. Several approaches exist for resecting esophageal cancer. In this study, neoadjuvant chemoradiotherapy consisting of 2–6 cycles was administered for patients who were not candidates for surgery. For such patients, surgery was considered if the size of the tumor decreased after neoadjuvant treatment. Specifically, surgery was conducted 4–8 weeks after completing neoadjuvant chemoradiotherapy.

Patients were comprehensively examined after hospital admission. Following the European Society for Clinical Nutrition and Metabolism (ESPEN) guidelines [12], patients received nutritional support in the presence of any of the following criteria: 1) weight loss of ≥10% within the last month; 2) body mass index (BMI) <18.5 kg/m^2^; and 3) serum albumin level less than 30 g/L in the absence of liver or kidney dysfunction as assessed by the subjective global assessment (SGA) grade C. Nutritional support was initiated 7 to 14 days before surgery. The nutritional support was provided via the combination of oral and intravenous routes, with an emphasis on intravenous supplementation. High-protein and high-calorie diets were preferred for oral supplementation. Artificial nutrients, such as glucose, amino acids, and lipids, were given intravenously. Specialized nutritionists adjusted the regimen if necessary. Patient's eligibility for surgery was reassessed 7 to 14 days after initiating the nutritional support.

### Surgical procedure

#### SPTE

**Fig. 1:**
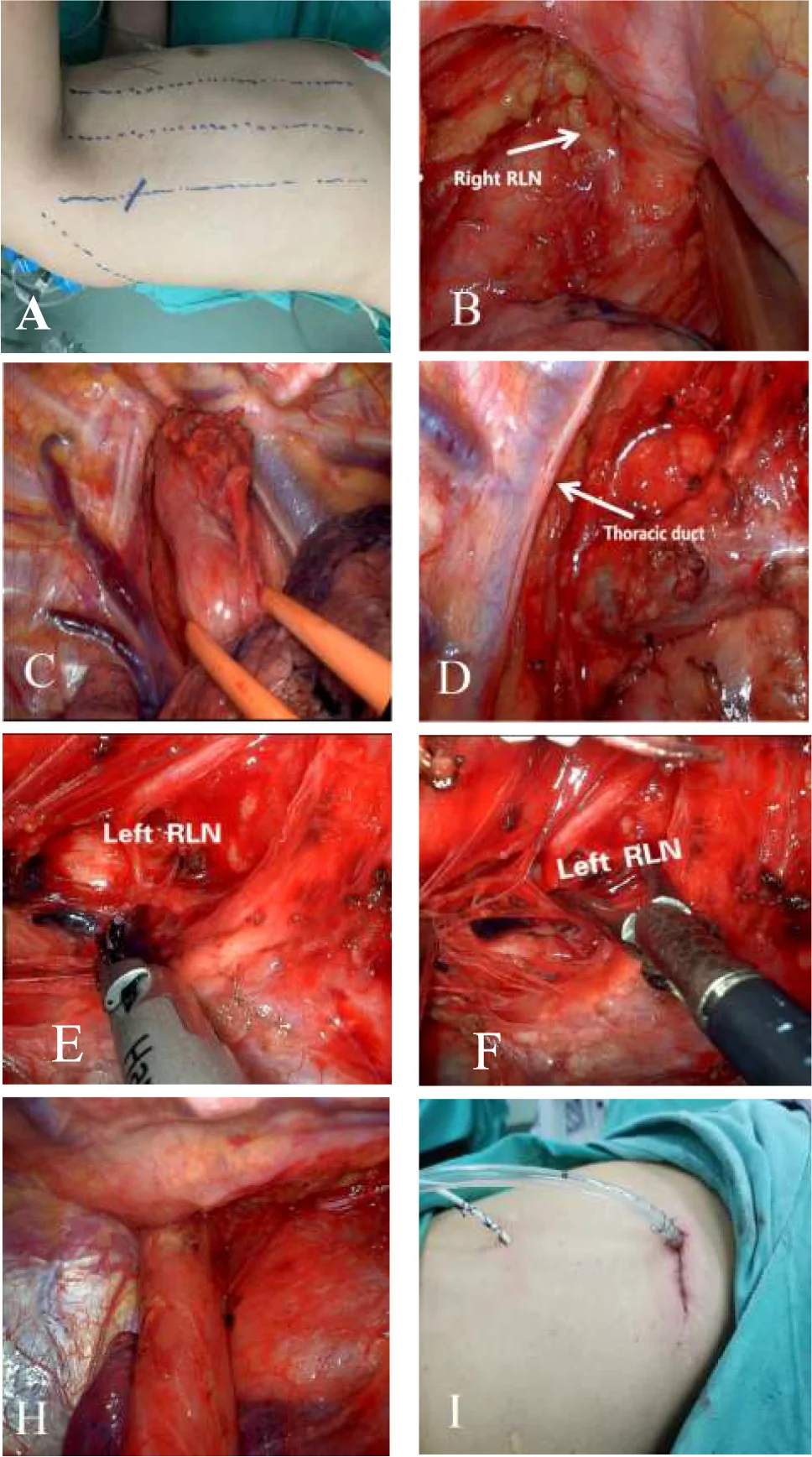
Different stages of SPTE

Patients were placed in the left lateral decubitus position, and surgeons were on the abdominal side. Based on the tumor location, a 3-cm incision was made on the fourth rib along the right posterior axillary line to access the thoracic cavity (Figure 1A). The innominate vein arch was mobilized and ligated after opening the mediastinal pleura. After ligating the innominate vein, the right recurrent laryngeal nerve near the right subclavian artery was exposed and the nerve integrity was preserved. The surgeon dissected lymph nodes around the right recurrent laryngeal nerve (Figure 1B). Thereafter, lymph nodes adjacent to the upper esophagus were sequentially dissected. The upper end of the esophagus was released and pulled using a urinary catheter or gauze (Figure 1C). At this stage, the surgeon attempted to preserve the thoracic duct (Figure 1D). The left recurrent laryngeal nerve was bluntly exposed after fully exposing the lymph nodes below the carina (Figure 1E). Thereafter, the lymph nodes adjacent to the left recurrent laryngeal nerve were dissected (Figure 1F). The middle and lower segments of the esophagus were released up to the esophageal hiatus (Figure 1H). Furthermore, the lymph nodes around the esophagus, pulmonary veins, and trachea were sequentially dissected. Drains were placed (Figure 1I), and the patient was then placed in the supine position.

#### FPTE

A 1 cm incision was made in the 7 th intercostal space of the right anterior axillary line, and a trocar was inserted as the entry hole to establish an artificial pneumothorax with CO2. A 1 cm incision was made in the 4 th intercostal space of the right midaxillary line, a 0.5 cm incision was made in the 5 th intercostal space, and a 1 cm incision was made in the 9 th intercostal space of the anterior edge of the subscapular angular line to implant trocar. They were used as the main and secondary operating holes. We slit the mediastinal pleura on the surface of the esophagus along the edge of the mediastinal surface of the lung and the space of the thoracic aorta of the esophagus. Thereafter, we released the esophagus down to the esophageal hiatus and simultaneously resected the paraesophageal lymph nodes, cut the mediastinal pleura along the upper margin of the azygos vein, cut the mediastinal pleura along the edge of the right vagus nerve and the surface of the right subclavicular artery, and released the right vagus nerve. Lymph nodes in the superior mediastinum around the esophagus and right parapharyngeal nerve were dissected. The azygos vein was dissociated and dissected, and the subcarina, and left and right parabronchial lymph nodes were dissected. The left recurrent laryngeal nerve was exposed and dissected by blunt and sharp separation along the trachea and left main bronchus. Above the diaphragm, the thoracic duct was released from the thoracic aorta and the medial azygos vein. A hemolock vascular clamp was used to close or protect the thoracic duct. Finally, a drain was placed.

#### Abdominal surgery

Five trocars were placed for abdominal surgery. Next, pneumoperitoneum was established. The right gastroepiploic artery was preserved along the greater omentum. Using a cutting stapler, a gastric tube was formed and then buried. To mobilize the cervical segment of the esophagus, a slanted incision was made in the left neck. The gastric tube was cut and pulled through the esophageal bed to the left neck. Subsequently, a gastroesophageal end-to-side anastomosis was conducted, and a cervical anastomosis was manually done.

### Follow-up and discharge

After the surgery, we closely followed the patients to ensure that: 1) patients exhibited good bilateral lung breathing sounds; 2) no gas leakage was observed in the chest tube; 3) drainage fluid had clear or non-chylous appearance with a volume of less than 100 mL per 24 h; 4) chest X-ray indicated satisfactory lung re-expansion, allowing for chest tube removal; 5) patients experienced no specific discomfort after receiving the semi-liquid diet; 6) patients did not suffer from fever, chest tightness, or shortness of breath; and 7) follow-up blood biochemical test results and gastrointestinal imaging showed no abnormalities, allowing for patients’ discharge. Patients with pT2-4 N1-2M0 who tolerated further treatment received adjuvant radiotherapy and chemotherapy after surgery [13].

### Data collection

The following data were collected as preoperative characteristics: age, sex, weight, BMI, tumor type, T stage, lymph node involvement, TNM stage, smoking, alcohol drinking, neoadjuvant chemoradiotherapy, and presence of diabetes, hypertension, cardiac disease (including heart failure and coronary heart disease), and chronic obstructive pulmonary disease (COPD). We also collected perioperative data and surgical outcomes. Surgical outcomes were as follows: 3-month, 6-month, and 12-month overall survival of patients, chest drainage length, duration of surgery, estimated intraoperative blood loss, time to initiating oral feeding, and length of postoperative hospital stay. Tumor staging was done following the 11^th^ edition of the Japan Esophagus Society (JES) staging system [14]. The following adverse events and complications were collected in this study: postoperative chylothorax, postoperative empyema, postoperative arrhythmia, postoperative *esophagotracheal* fistula, recurrent laryngeal nerve injury, postoperative gastrointestinal symptoms, postoperative intractable pain, second surgery, and postoperative pneumonia (based on the definition of the Utrecht Pneumonia Score) [15].

We measured postoperative intractable pain using the standardized scoring scales for pain intensity, pain-related distress, and functional impairment for at least three months after the surgery defined by the International Classification of Functioning, Disability and Health [16].

### Statistical analysis

Data used for this study were analyzed using SPSS version 26.0 (IBM Corp, Armonk, NY, USA), and graphs were visualized using GraphPad Prism version 8.0 (GraphPad Software, San Diego, CA, USA). Qualitative data were compared using the chi-squared test, and are presented as numbers (percentages). The normal distribution of quantitative data was assessed using the Kolmogorov–Smirnov test. In the presence of normal distribution, the student’s t-test was used to compare the two groups; otherwise, the Mann–whitney U test was used to compare the two groups. Quantitative data with normal distribution are presented as mean ± standard deviation (SD) and those without normal distribution are presented as median (interquartile range). The statistics reported in the following tables are assumed to come from normally distributed data unless otherwise mentioned. P-values less than 0.05 were considered statistically significant. Due to the importance of T staging and TNM staging on patients’ outcomes, we also conducted the one-way analysis of covariance (ANCOVA) test for all outcomes to partly attenuate the effects of these variables on comparisons.

## Results

### Baseline characteristics of patients

In total, 86 patients with esophageal cancer who underwent SPTE and 114 patients who underwent FTEP at the First Affiliated Hospital of Kunming Medical University from February 2019 to November 2022 and did not have any of the exclusion criteria were enrolled in this study. The preoperative characteristics of patients were comparable between the two groups (Table 1). The age of patients was 59.93 ± 7.12 years in the SPTE and 57.05 ± 9.02 years in the FPTE group. The majority of patients were male in both groups (90.7% in the SPTE group and 86.8% in the FPTE group). The number of comorbidities and the prevalence of major comorbidities, such as diabetes, hypertension, chronic obstructive pulmonary disease (COPD), and cardiac diseases, were comparable between the two groups. In total, 74.4% of patients in the SPTE group and 68.4% of patients in the FPTE group were smokers. Regarding the tumor type, pathological assessment indicated that most patients included in this study had esophageal squamous cell carcinoma. Furthermore, there was no significant difference between the two groups in terms of tumor type, neoadjuvant chemoradiotherapy, lymph node involvement, T staging, and TNM staging (Table 1).

**Table 1:** The baseline and preoperative characteristics of patients

		SPTE group (*n* = 86)	FPTE group (*n* = 114)	*P*-value
Age (year)		59.93 ± 7.12	57.05 ± 9.02	0.054
Male gender		78 (90.7%)	99 (86.8%)	0.072
Weight (kg)		59.16 ± 7.72	61.39 ± 7.72	0.068
BMI (kg/m^2^)		21.24 ± 2.89	21.63 ± 2.70	0.333
Number of comorbidities		1.18 ± 1.41	1.11 ± 1.14	0.716
COPD		22 (25.5%)	27 (23.7%)	0.720
Diabetes		23 (26.7%)	33 (28.9%)	0.826
Hypertension		26 (30.2%)	30 (26.3%)	0.487
Cardiac disease (heart failure or ischemic heart disease)		16 (18.6%)	18 (15.8%)	0.616
Smoking		64 (74.4%)	78 (68.4%)	0.295
Alcohol drinking		73 (84.8%)	84 (73.7%)	0.054
Tumor type (squamous cell carcinoma)		80 (93.0%)	99 (86.8%)	0.202
Neoadjuvant treatment		12 (13.9%)	15 (13.2%)	0.932
Lymph node involvement		41 (47.6%)	62 (54.4%)	0.305
T stage	1	11 (12.8%)	4 (3.5%)	0.081
	2	18 (20.9%)	24 (21.1%)	
	3	48 (55.8%)	69 (60.5%)	
	4	9 (10.5%)	17 (14.9%)	
TNM stage	1	13 (15.1%)	7 (6.1%)	0.110
	2	38 (44.2%)	52 (45.6%)	
	3	34 (39.5%)	55 (48.2%)	
	4	1 (1.2%)	0 (0.0%)	

Qualitative data are presented as numbers (percentages). *P*-values for comparing qualitative data were obtained from the chi-squared test. Quantitative data are presented as mean ± SD. *P*-values for comparing quantitative data were obtained from the student’s *t*-test. *BMI* body mass index, *COPD* chronic obstructive pulmonary disease, *FPTE* four-port thoracoscopic esophagectomy, *SPTE* single-port thoracoscopic esophagectomy

### Comparison of the surgical and postoperative outcomes between the two groups

The overall duration of surgery was significantly longer in the SPTE group (346.34 ± 53.86 min) than in the FPTE group (332.89 ± 28.86 min). Although the length of thoracic surgery did not significantly differ between the two groups (162.89 ± 37.00 min in the SPTE group vs. 166.84 ± 17.22 in the FPTE group), abdominal surgery significantly lasted longer in the SPTE group (183.46 ± 33.33 min) than in the FPTE group (166.05 ± 22.07 min) (Table 1).

Regarding postoperative overall survival, there was not a significant difference between SPTE and FPTE groups in terms of 3-month overall survival rate (100% vs. 97.2%, respectively), whereas 6-month (98.8% vs. 92.3%) and 12-month overall survival rates (96.5% vs. 86.8%) were significantly greater in the SPTE group (Table 2). Although the difference in overall survival became statistically non-significant after conducting the one-way ANCOVA test, suggesting that the difference in overall survival was largely driven by differences in T stage and TNM stage (Table 1 and Table 2).

**Table 2:** Surgical and postoperative outcomes of the two groups

	SPTE group (*n* = 86)	FPTE group (*n* = 114)	*P*-value before adjustment	*P*-value after adjustment for T stage and TNM stage
Overall survival after surgery for 3 months	86 (100%)	140 (97.2%)	0.141	0.167
Overall Survival after surgery for 6 months	85 (98.8%)	133 (92.3%)	0.038	0.069
Overall Survival after surgery for 12 months	83 (96.5%)	125 (86.8%)	0.025	0.065
Time to start oral feeding (day)	9.36 ± 3.05	9.24 ± 2.06	0.121	0.657
Chest drainage length (day)	6.06 ± 2.16	7.45 ± 1.68	0.000	0.000
Length of hospital stay after surgery (day)	13.54 ± 3.88	19.08 ± 5.09	0.000	0.000
Estimated intraoperative blood loss (mL)	226.05 ± 94.87	363.16 ± 78.92	0.000	0.000
Thoracic surgery time (min)	162.89 ± 37.00	166.84 ± 17.22	0.373	0.79
Abdominal surgery time (min)	183.46 ± 33.33	166.05 ± 22.07	0.000	0.000
Overall length of surgery (thoracic + abdominal) (min)	346.34 ± 53.86	332.89 ± 28.86	0.043	0.000

Data are presented as numbers (percentages) or mean ± SD. *P*-values were obtained from the chi-squared test for qualitative data and the student’s *t*-test for quantitative data. *FPTE* four-port thoracoscopic esophagectomy, *SPTE* single-port thoracoscopic esophagectomy

**Fig. 2:**
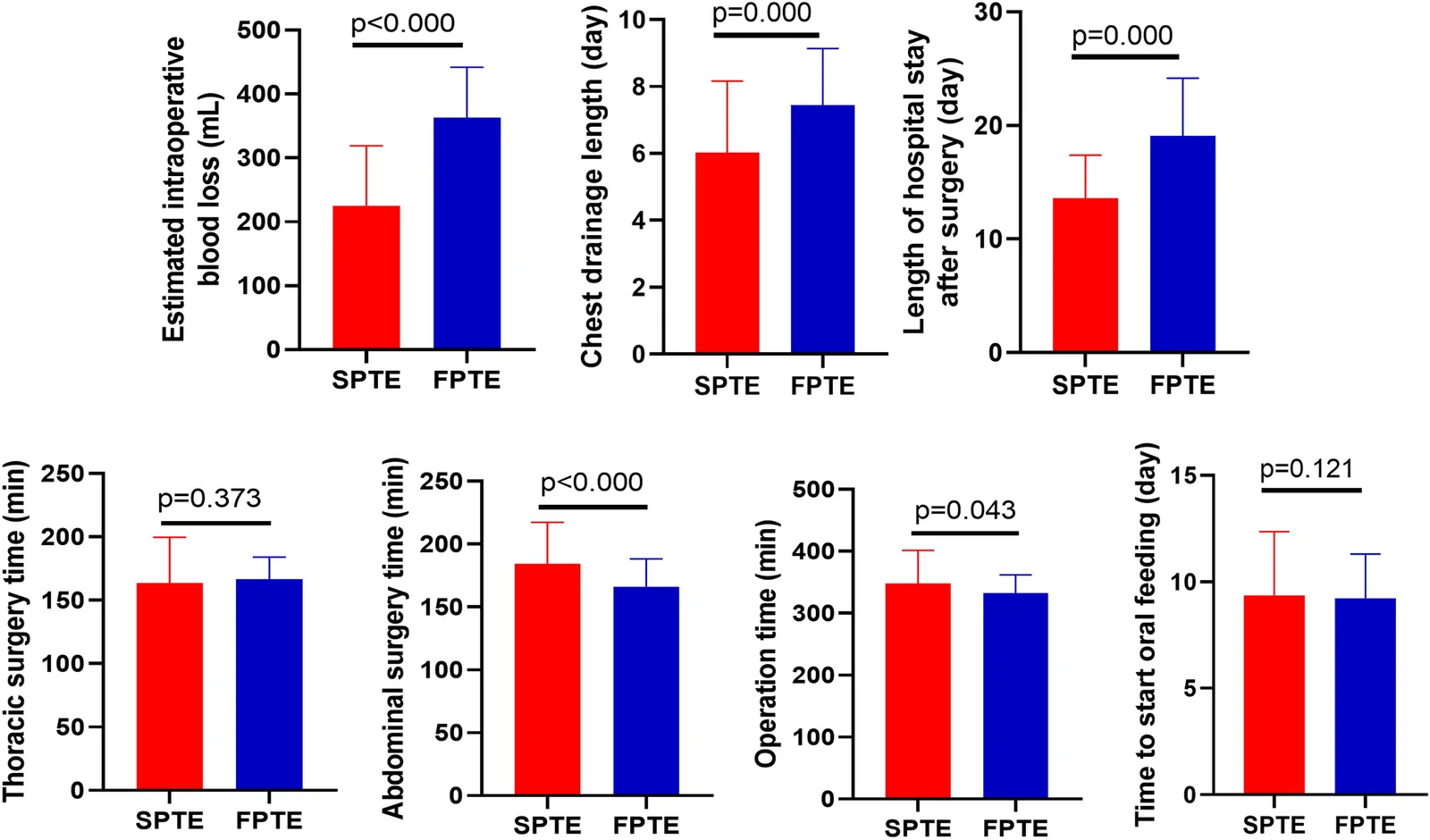
Comparison of some of the surgical outcomes between the two groups. Error bars show SD

The duration of Chest drainage was significantly shorter in the SPTE group (6.06 ± 2.16 days), compared to the FPTE group (7.45 ± 1.68 days). Similarly, the length of hospital stay after esophagectomy was significantly shorter in the SPTE group (13.54 ± 3.88 days), compared to the FPTE group (19.08 ± 5.09 days) (Table 2 and Fig. 2).

Estimated intraoperative blood loss was significantly lower in the SPTE group (226.05 ± 94.87 mL) than in the FPTE group (363.16 ± 78.92 mL). However, there was no significant difference between the SPTE group (9.36 ± 3.05 days) and the FPTE group (9.24 ± 2.06 days) in terms of the time to start oral feeding (Table 2 and Fig. 2).

### Comparison of postoperative complications between the two groups

Intractable pain lasting for 3 months postoperatively was significantly more common in the FPTE group (39.5%) than in the SPTE group (21.0%); however, the pain gradually resolved in more than half of patients and the incidence rate of intractable pain lasting for 6 months (14.0% in the SPTE group vs. 21.1% in the FPTE group) and 12 months (11.6% in the SPTE group vs. 14.9% in the FPTE group) did not significantly differ between the two groups (Table 3).

**Table 3:** Postoperative complications of the two groups

	SPTE group (*n* = 86)	FPTE group (*n* = 114)	*P*-value before adjustment	*P*-value after adjustment for T stage and TNM stage
Intractable pain after surgery for 3 months	18 (21.0%)	45 (39.5%)	0.006	0.008
Intractable pain after surgery for 6 months	12 (14.0%)	24 (21.1%)	0.180	0.526
Intractable pain after surgery for 12 months	10 (11.6%)	17 (14.9%)	0.499	0.788
Postoperative pneumonia	10 (11.6%)	39 (34.2%)	0.000	0.000
Recurrent laryngeal nerve injury	11 (12.7%)	9 (7.9%)	0.197	0.068
Postoperative gastrointestinal symptoms	36 (41.9%)	27 (23.7%)	0.007	0.002
Postoperative chylothorax	2 (2.3%)	12 (10.5%)	0.032	0.048
Postoperative empyema	3 (3.5%)	17 (14.9%)	0.007	0.039
Postoperative arrhythmia	7 (8.1%)	15 (13.2%)	0.326	0.404
Postoperative *esophagotracheal* fistula	3 (3.5%)	3 (2.6%)	0.669	0.980
Second surgery	2 (2.3%)	6 (5.3%)	0.332	0.165

Data are presented as numbers (percentages). *P*-values were obtained from the chi-squared test. *FPTE* four-port thoracoscopic esophagectomy, *SPTE* single-port thoracoscopic esophagectomy

Postoperative pneumonia was a frequent complication with a significantly higher incidence rate in the FPTE group (34.2%) than in the SPTE group (11.6%). Similarly postoperative empyema (14.9% vs. 3.5%) and postoperative chylothorax (10.5% vs. 2.3%) were significantly more common in the FPTE group than in the SPTE group. In contrast, postoperative gastrointestinal symptoms were significantly more common in the SPTE group (41.9%) than in the FPTE group (23.7%) (Table 3).

Recurrent laryngeal nerve injury (12.7% vs. 7.9%) and postoperative *esophagotracheal* fistula (3.5% vs. 2.6%) were partly more common in the SPTE group, and postoperative arrhythmia (13.2% vs. 8.1%) and second surgery (5.3% vs. 2.3%) were more common in the FPTE group; however, none of them met the threshold of statistical significance (Table 3).

## Discussion

This study retrospectively compared the clinical data from 86 patients with esophageal cancer who underwent McKeown SPTE and 114 patients with esophageal cancer who underwent FPTE. Compared to patients undergoing FPTE, patients undergoing SPTE had shorter hospital stay and chest drainage, lower estimated intraoperative blood loss, and longer length of overall surgery and abdominal surgery. However, 3-month, 6-month, and 12-month overall survival, time to start oral feeding, and length of thoracic surgery were comparable between the two groups. In addition, intractable pain lasting for 3 months and postoperative pneumonia, empyema, and chylothorax were significantly less frequent among patients undergoing SPTE, whereas postoperative gastrointestinal symptoms were less frequent in the FPTE group.

In a single-center study, Lee et al*.* compared the outcomes of 315 patients who underwent traditional minimally invasive esophagectomy and 15 patients who underwent single-port minimally invasive esophagectomy for esophageal cancer [17]. Contrary to our findings, they reported no significant difference between the two groups in terms of blood loss, hospital stay, and operation time [17]. Another study with 48 patients in the single-incision and 48 patients in the multi-incision minimally invasive esophagectomy for esophageal cancer reported no significant difference between the two groups in terms of postoperative ICU and hospital stay and the presence of major surgical complications, such as anastomotic leakage and pulmonary complications [18]. In that study, the pain score was significantly lower in the single-incision group after one week of the surgery [18]. Similarly, we found that patients in the SPTE group were less likely to experience intractable pain lasting for 3 months.

A study with 134 patients undergoing single-port VATS and 188 patients undergoing multi-port VATS for lung cancer indicated that the operation time and postoperative hospital stay were significantly longer in the multi-port VATS group [19]. Furthermore, intraoperative bleeding volume was significantly higher in the multi-port VATS group [19].

Consistent with our findings, a meta-analysis of 39 observational studies with 4635 patients undergoing lung resection or other intrathoracic procedures indicated that postoperative pain, blood loss, length of postoperative stay, and chest drainage duration were significantly lower in the single-incision VTAS group compared to the multiple-incision VTAS group [20].

Compared to open esophagectomy, minimally invasive esophagectomy has significantly decreased the incidence rate of complications, such as pulmonary complications, infections, and bleeding [21]. However, pulmonary complications, such as pneumonia and empyema, dysrhythmias, anastomosis leakage, recurrent laryngeal nerve injury, gastrointestinal complications, and chyle leakage are still some of the most frequent complications of minimally invasive esophagectomy [4, 22–25]. The incidence rate of such complications in minimally invasive esophagectomy has been reported to be more than 10%−20% in some studies [23, 25]. Thus, there is still space for improvement in this regard. SPTE via the McKeown esophagectomy approach is safe and feasible, with few manageable complications [26]; however, a few studies set out to compare its efficacy with FPTE. This study comprehensively compared postoperative complications between SPTE and FPTE and found that most of these complications, such as pneumonia, empyema, and chylothorax were more commonly observed in the FPTE group, and only postoperative gastrointestinal symptoms were significantly more common in the SPTE group. The duration of surgery, the volume of blood loss, the length of hospital stay, and the complication rates after SPTE widely vary across different studies and highly depends on the expertise of the surgeons [25, 27–29].

The advent of a robotic single-port system can also achieve more favorable outcomes, shorten the length of surgery, and decrease the risk of complications [30–32]. For instance, a meta-analysis reported that compared to conventional minimally-invasive McKeown esophagectomy, robot-assisted minimally-invasive esophagectomy can decrease the incidence of lung complications and hospital stay and increase the number of harvested lymph nodes [32]. Interestingly, a study comprising 41 patients undergoing single-port mediastinoscopic radical esophagectomy with mediastinal lymphadenectomy reported that continuous nerve monitoring for recurrent laryngeal nerve significantly decreased the incidence of postoperative recurrent laryngeal nerve palsy (4.0% vs. 31.2%) and postoperative pneumonia (4.0% vs. 18.8%) and markedly shortened the length of postoperative hospital stay (13 days vs. 41 days) (33). Such modifications to the surgical approach are believed to further decrease the risk of complications and enhance patients’ safety.

## Limitations

There are several limitations to our study that must be acknowledged. First, this study was a single-center study and included a limited number of participants. Future multi-center studies with larger sample sizes are needed to provide more robust evidence and enhance the generalizability of findings. Second, the retrospective design of this study might have increased the risk of bias. Compared to retrospective studies, prospective studies are less susceptible to bias and provide more reliable findings. In particular, randomized controlled trials can minimize the risk of bias to a greater extent. Third, as the time-to-event variable was not measured for survival length, we could not conduct a survival analysis and compare the survival of patients with time passage more accurately and serially. Fourth, expert surgeons with extensive experience in minimally invasive esophagectomy conducted surgeries in this study, but surgeons’ experience is a critical factor affecting the outcome of surgery and postoperative complications in such highly specialized surgeries. Due to these limitations, the results of this study must be interpreted with caution.

## Conclusions

Our findings revealed that patients undergoing SPTE had shorter hospital stay and chest drainage, and lower estimated intraoperative blood loss. In addition, intractable pain lasting for 3 months and postoperative pneumonia, empyema, and chylothorax were significantly less frequent among patients undergoing SPTE. In contrast, the overall length of surgery and the length of abdominal surgery were shorter among patients undergoing FPTE. Furthermore, postoperative gastrointestinal symptoms were less frequent in the FPTE group.

## Data Availability

Data will be available from the corresponding author on a reasonable request.
